# Invisible Blood Corpuscles

**Published:** 1884-01

**Authors:** J. M. Adams


### INVISIBLE BLOOD CORPUSCLES.

J. M. ADAMS.

It is rather remarkable that more has not been said about the "invisible blood corpuscles" discovered by Dr. Norris, of Manchester, England, several years ago. Changing or increasing the density of the blood by adding sugar or other substances, [with or without staining,] from three to five times as many delicately clear, smooth, transparent and colorless corpuscles appear as there are red and white corpuscles together, as ordinarily seen without this preparation.

These "ghost-like," very flat, hyaline corpuscles, almost filling the liquor sanguinus and serum, were first revealed, strange to say, by the photograph plate, and since then, many of these plates have been made by the discoverer mentioned, showing that these so-called "invisible corpuscles" exist in the blood of all animals as well as man, and furthermore that a great similarity of form and size exists in all the species examined, although the other corpuscles greatly differ.

Their size and shape are about the same as the ordinary red corpuscles in man, although averaging perhaps a little smaller and thinner. They contain no hæmatin or visible contents; no nucleus, and very evidently are not red corpuscles, rendered transparent by endosmosis, for they never appear distended or engorged, as usually seen in this case, nor are they old, worn-out corpuscles, for they are never ragged-edged, broken or mutilated in any manner; but, on the contrary, appear to be freshly formed. They do not resemble in any respect the white corpuscle or blood leucocytes, being much more transparent, flatter, smaller, never granular, and acting differently with stainings, solvents and re-agents. They do not materially change in form by endosmosis or exosmosis, and in fact do not seemingly take up or give off oxygen or carbonaceous products. They are not like lymph or chyle corpuscles, which are spheroidal, granular and of immature appearance, much less like the mucous or any other corpuscle known, but distinct, separate and peculiar, and unless they be transitional forms somehow not yet understood, they must be evidently a third corpuscular element in the blood.

They are very easily affected by any agent causing coagulation, and seem to form a large part of the fibrine. Whether or not they are easily affected by zymotic or contagious influences, or perform any specific work other than coagulation, is a question undecided; but it must be allowed that some very important purpose is served by them, being so much more numerous than the other corpuscles, and herein a new field of remarkable interest is opened before the microscope.

Truly the deep mysteries of life are being slowly revealed; not, however, except by searching, untiring and patient labor, which every microscopist should be willing to give, for no one investigator, situated as favorably as he may be, is able to do this great work alone, besides correcting the errors that may arise by looking at the subject from one standpoint; nevertheless much is yet to be known, and the motto of every microscopist should be, "deeper, down deeper, and wider, still wider," no matter what field he may happily explore.

Commenting upon the foregoing, we desire to say that much has been learned of the blood corpuscles, within a few years, by means of the microscope, that had not been before suspected. So thoroughly has the blood been inspected that it has been found possible, by many observers, to distinguish between the blood of man and that of animals. Indeed, microscopists have been found who will even undertake to name the animal from which a specimen of blood has been taken. This we have known to have been successfully accomplished.—[ED. BISTOURY.]

